# Dual TCR-Expressing T Cells in Cancer: How Single-Cell Technologies Enable New Investigation

**DOI:** 10.4049/immunohorizons.2200062

**Published:** 2023-05-02

**Authors:** Elizabeth M. Muhowski, Laura M. Rogers

**Affiliations:** Department of Immunology, Mayo Clinic, Rochester, MN; Department of Immunology, Mayo Clinic, Rochester, MN

## Abstract

TCR diversity measures are often used to understand the immune response in cancer. Traditional measures of diversity rely on bulk RNA sequencing (RNAseq) of the β-chain variable regions. However, the full αβ TCR repertoire is a combination of both the α- and β-chains, which are encoded by separate genes. In contrast with bulk RNAseq, single-cell RNAseq (scRNAseq) allows paired chain analyses, yielding a more accurate measure of the repertoire. Interestingly, **∼**30% of mature peripheral T cells express multiple TCR alleles (e.g., two α-chains) and may exhibit dual Ag specificity. scRNAseq has become increasingly common, and data from both human and animal studies are publicly available. However, routine workflows discard secondary TCR alleles and focus on a single TCR clone per cell. This perspectives piece emphasizes why this may not be good practice and highlights unanswered questions in the field of T cell dual specificity.

## Single-cell technologies allow us to observe the TCR repertoire with greater resolution

Cancer immunotherapies, including cancer vaccines, immune checkpoint blockades, oncolytic virus, cytokine, and adoptive cell transfer therapies, have fundamentally changed the treatment strategy for many cancer types (1). The goal of immunotherapy is to induce or improve the ability of the immune system to recognize and eliminate cancer cells. Using the immune system to kill tumor cells means that treatment is specific, dynamic, and potentially more durable compared with traditional targeted therapies. However, immunotherapy has significant drawbacks: serious immune-related toxicities are commonplace (2), financial burden can be substantial, and predicting which patients will respond remains difficult (3). Thus, cancer immunology has become a major focus of cancer research as scientists try to better understand the heterogeneous tumor microenvironment (TME) and the changes that take place over time and in response to therapies. Often, the focus is on connecting various aspects of T cell antitumor responses, such as T cell phenotyping for exhaustion level (intratumoral and peripheral) or TCR repertoire diversity with patient response to immunotherapy (4, 5).

The TCR is the basis by which T cells recognize Ag peptides presented by MHCs. Tumor cell–intrinsic mutations can produce recognizable tumor Ags, which can lead to the clearance of mutated tumor cells through immunosurveillance (6). Understanding the clonally expanded TCR repertoire supports identification of strong tumor Ags for which cancer vaccines may be generated. A greater diversity of TCR clonotypes in a repertoire means that a greater diversity of Ags may elicit T cell responses, and lower TCR diversity in the TME correlates with worse prognosis in patients (4).

Burnet’s clonal selection theory posits that one T cell expresses a single somatically recombined TCR sequence on the T cell surface despite a diploid genome (7). During T cell development in the thymus, the TCR gene locus undergoes genetic rearrangement and subsequent allelic exclusion to produce a single TCR. For αβ T cells, the β-chain genes (including the commonly sequenced *TRBV*) rearranges first during the double-negative stage (8). Once a β-chain V-D-J recombination produces a protein that propagates a pre-TCR signal, the cell suppresses rearrangement and expression of any remaining β-chain allele by degrading the RAG recombinase and chromatin remodeling of the remaining *TRBV* locus. If no functional β-chain is produced (i.e., nonproductive TCR rearrangement), the cell undergoes apoptosis. Later, at the double-positive stage, both α-chain alleles rearrange simultaneously (9, 10), and rearrangement continues until the cell completes positive thymic selection, ensuring that the T cell can bind MHC. With imperfections in allelic exclusion of TCR β alleles and in functional exclusion of TCR α-chains, mature T cells expressing multiple TCR alleles at the mRNA and protein levels have been observed in the periphery of both mice and humans (11).

Recent technological advances in single-cell RNA sequencing (scRNAseq) allow direct measurement of cells expressing multiple TCR alleles at the mRNA level, as well as simultaneous characterization of gene expression in individual cells (12, 13). Because transcripts from individual cells are labeled with a unique barcode, scRNAseq enables observation of *TRAV* and *TRBV* expression within an individual cell to allow more accurate measures of TCR diversity. Currently, the accuracy of scRNAseq TCR diversity measures is limited by issues such as empty droplet or doublet captures where transcripts appear to derive from a single cell when, in fact, they derive from noncellular debris or multiples. In addition, scRNAseq generally suffers from low sampling depth, such that fewer cells are sampled in single-cell workflows than in bulk sequencing. In response to these issues, computational models have been developed that aim to enumerate dual TCR-expressing cells more accurately from scRNAseq data (14). As throughput of single-cell technologies grows, sampling depth may not pose a challenge for long.

Because of the differences in mechanisms of allelic exclusion during thymic development described above, mature T cells in the periphery are more likely to express two α-chains than two β-chains. Indeed, up to 30% of human peripheral T cells express two rearranged *TRAV* alleles at the mRNA level (15). Accurate estimates of dual TCR expression at the protein level are challenging because of a paucity of Ab reagents against different V segments; however, current estimates suggest that 10% of mature T cells express two α-chains, and 1–7% express dual β-chains on the cell surface (11, 15–20). Importantly, T cells that express dual TCRs on the cell surface can propagate signal through either receptor and could expand the TCR repertoire diversity (21, 22). Given the potential utility of measuring TCR diversity over time in patients with cancer and the increasing use of single-cell technologies to track this, it is striking that most literature on the subject neither quantifies nor discusses dual TCR-expressing cells in the TME.

## Dual TCR-Expressing Cells Are Functionally Distinct from Single TCR-Expressing Cells

Since their discovery, dual TCR-expressing cells have been hypothesized to contribute to autoimmunity based on the mechanism of thymic selection during T cell development. For example, during the double-positive stage of T cell development, where both α-chain alleles are recombined simultaneously, the α-chains that successfully pair with the β-chain are expressed on the cell surface and evaluated for MHC binding affinity (positive selection) (23). If both α-chains productively pair with the β-chain, only one needs to effectively bind MHC to pass through positive selection. Because of this, the second TCR is not required to be MHC restricted and may exhibit novel Ag specificities that are auto- or alloreactive (22, 23).

In models of autoimmunity, dual TCR-expressing cells are not always primary drivers via autoreactive Ag specificities (24), and, in some cases, dual TCR-expressing cells contribute to autoimmune pathology by limiting regulatory T cell development (25). In humans, clonally expanded dual TCR cells have been observed in active psoriatic plaques and multiple sclerosis lesions (26), and cells with dual TCR specificities against autoantigens and viral Ags can become actively autoreactive upon viral Ag exposure (27, 28). Thus, dual TCR-expressing autoreactive peripheral T cells may be tolerized until activation through the second, nonautoreactive TCR breaks this tolerance. Although potentially detrimental in autoimmunity, T cells with dual specificity may be an advantage in antitumor immunity, where tumor-associated Ags only weakly activate autoreactive T cells (29). Lower avidity tumor-associated Ag–specific T cells can positively contribute to antitumor immunity (30), but these cells may exhibit stronger antitumor function if activated through a second TCR.

However, patients with cancer receiving immunotherapy frequently develop immune-related toxicities that are often autoimmune in nature (31). In this case, dual TCR-expressing cells may contribute to immunotherapy toxicity to the detriment of the patient. Furthermore, adoptive transfer of TCR-engineered T cells that retained expression of their endogenous TCR resulted in dual specific T cells with lower target avidity, possibly due to unintended pairing of exogenous and endogenous α/β-chains (32). Because unintended pairings can also produce unpredictable Ag specificities, authors instead replaced the endogenous TCR with the engineered TCR and found reduced off-target reactivity after adoptive transfer. It will be highly important to study whether dual TCR-expressing cells play direct, functional roles in immunosurveillance and immunotherapy-induced toxicities.

Dual TCR-expressing cells also appear functionally distinct in response to foreign Ags. Two independent studies have observed that dual TCR expressors were not expanded in the naive repertoire, but preferentially expanded upon exposure to foreign Ag (22, 33). Similarly, dual TCR-expressing cells are significantly overrepresented in the VDJdb database (https://vdjdb.cdr3.net/) of known Ag-specific TCR sequences, suggesting preferential expansion of potentially dual-reactive T cells upon Ag exposure (14). Importantly, dual TCR-expressing cells in viral infection are both preferentially expanded and more likely to persist as immunologic memory after viral clearance (33). By extension, we might anticipate that dual TCR-expressing cells could have greater immunosurveillance capabilities during cancer development and upon immunotherapy administration than single TCR-expressing cells.

Exogenously engineered dual-specificity cells have been used in adoptive transfer therapy. Engineered T cells expressing an endogenous TCR against a nontumor Ag and an exogenously introduced chimeric Ag receptor (CAR) against a tumor Ag demonstrated better tissue infiltration, expansion, and memory formation than single-specificity T cells in independent studies (34, 35). However, dual-reactive cells expressing one TCR and one CAR are not necessarily equivalent to dual-reactive T cells expressing two TCRs. Most dual TCR cells result from expression of both α-chain alleles that competitively pair with a single expressed β-chain (21, 36), which can result in reduced antitumor control (37).

Taken together, it is difficult to predict whether endogenous dual TCR-expressing T cells would correlate with better or worse prognosis. Furthermore, prognostic correlations may be dependent on whether and what immunotherapy is administered. Additional studies are needed to clarify whether prognostic correlations exist and indeed whether dual TCR-expressing cells are preferentially expanded in cancer as in virus-infected tissue. Single-cell sequencing datasets are highly useful to begin exploring these ideas.

## Endogenous Dual TCR-Expressing Cells in Human Cancer

As previously noted, TCR diversity in the TME correlates with cancer prognosis (4); yet, information about dual expressing TCRs in cancer is extremely underreported in the literature. Traditional methods of quantifying dual TCR-expressing T cells rely on Abs directed toward specific *TRAV* or *TRBV* gene products and observing those cells bound by two different Abs (38). This is not only low throughput but also severely limited by Ab availability. scRNAseq is decidedly higher throughput; however, most analysis pipelines exclude consideration of the second expressed allele of the α or β-chain, keeping only the sequences with highest expression values. Indeed, reanalysis of a small peripheral blood dataset from healthy donors revealed that TCR diversity was significantly underestimated when dual TCR sequences were excluded from analysis (14).

This practice may not be biologically justified, given that evidence exists to support the idea that dual expressing T cells may respond to immunologic challenge differently from single TCR-expressing cells. For example, viral infection with influenza or lymphocytic choriomeningitis virus results in greater expansion of T cells expressing multiple TCR alleles over time than single TCR expressors (33, 39). Curiously, one study also observed enhanced expansion of T cells expressing two *TRAV* alleles even when one of the alleles was out of frame, though the value of retaining expression of an out-of-frame *TRAV* transcript is unclear (39). Furthermore, dual TCR expressors were clonally amplified in memory pools to a greater extent than single TCR expressors, supporting a functional distinction between single and dual expressors (33). Thus, it is important to understand whether dual TCR expressors are present in cancer tissues and whether they are differentially expanded.

To compute the frequencies of dual TCR-expressing T cells in cancer and to explore clonal expansion across tissues, we reanalyzed a recently published pan-cancer single-cell atlas with gene expression and TCRseq data from 87 treatment-naive patients with cancer and 15 cancer types (40). These studies include matched peripheral blood, tumor tissue, and normal tumor-adjacent tissue from each individual. TCR sequences and clonality measures were calculated by original authors using STARTRAC (40, 41). From their dataset, we split T cells into three groups: those expressing two *TRAV* alleles and one *TRBV* allele (“Dual TCRα”; *n* = 15,754 cells), those expressing one *TRAV* allele and two *TRBV* alleles (“Dual TCRβ”; *n* = 5,381 cells), and those expressing one *TRAV* and one *TRBV* allele (“Single TCR”; *n* = 144,839 cells).

**FIGURE 1.**
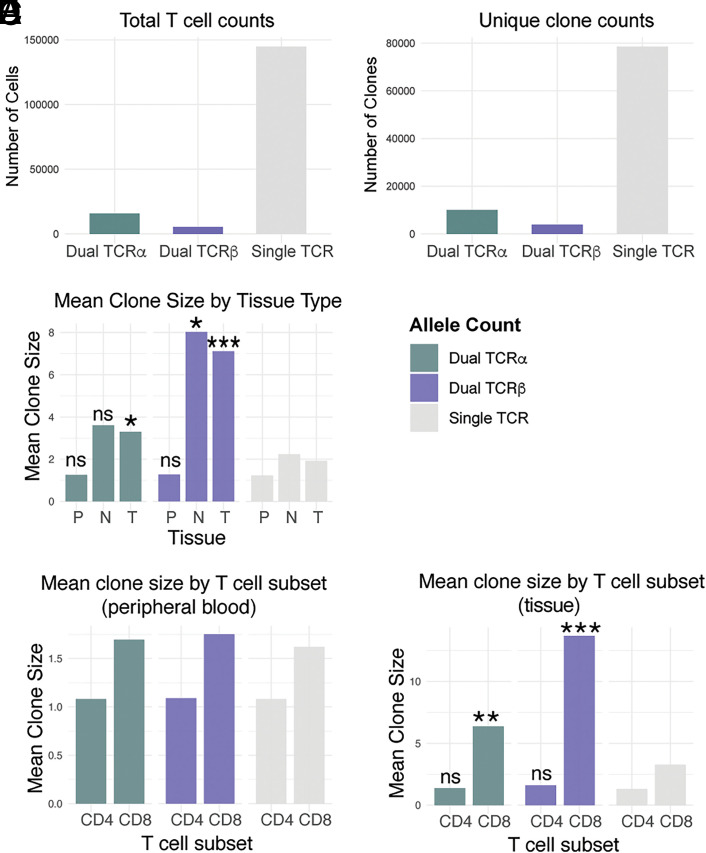
Frequency and clonal expansion of dual TCR-expressing T cells in cancer. (**A**) There are a total of 168,901 cells with TCRs across all samples. Of these, 85.8% are expressing a single *TRAV* allele and a single *TRBV* allele inframe (single TCR). Of the remaining 14.2% of cells expressing more than one *TRAV* or *TRBV* allele, the majority of these expressed two *TRAV* alleles and one *TRBV* allele (Dual TCRα, 9.3%), whereas 3.2% expressed one *TRAV* allele and two *TRBV* alleles (Dual TCRβ). This is consistent with prior observations where coexpression of two *TRAV* alleles was more frequently observed than coexpression of two *TRBV* alleles. Cells expressing two *TRAV* and two *TRBV* alleles (1.7% of cells in dataset) were excluded because it was not possible to determine whether these were doublets that made it through initial quality control settings or true expression values. (**B**) There are 95,102 unique TCR clones from 168,901 total T cells in this dataset. Of the unique clones, 82.6% are expressing a single *TRAV* allele and a single *TRBV* allele inframe (single TCR). Of the remaining 17.4% of unique clones expressing more than one *TRAV* or *TRBV* allele, the majority of these (10.6% of unique clones) are expressing two *TRAV* alleles and one *TRBV* allele (Dual TCRα). (**C**) The mean clone size of dual expressing T cells (Dual TCRα, teal; Dual TCRβ, purple) compared with single expressing T cells (Single TCR, gray) was significantly larger in tissues (normal adjacent, N; or tumor, T) compared with peripheral blood (P). (**D**) The mean clone size of dual expressing T cells (Dual TCRα, teal; Dual TCRβ, purple) compared with single expressing T cells (Single TCR, gray) was the same for both CD4^+^ and CD8^+^ subsets in the peripheral blood. (**E**) The mean clone size of dual expressing T cells (Dual TCRα, teal; Dual TCRβ, purple) compared with single expressing T cells (Single TCR, gray) was the same for CD4^+^ subset in tissue. However, mean clone size was significantly larger in CD8^+^ subsets. Significance for all panels was determined by Welch’s ANOVA with Games-Howell correction (*p < 0.05, **p < 0.01, ***p < 0.001).

Prior studies found that ∼30% of the T cells in normal human peripheral blood express more than one rearranged *TRAV* or *TRBV* allele at the mRNA level, and ∼10% of T cells express dual TCRs at the protein level on the cell surface (15–18). The proportions of cells across all tissues in the Zheng dataset expressing dual *TRAV* or dual *TRBV* alleles were 12.7% and 3.2%, respectively (Figure 1A). As expected, the unique clone counts in each group were lower than the total number of cells (Figure 1B), and the proportions of unique clones across all tissues expressing dual *TRAV* or dual *TRBV* mRNA were 10.9% and 4.2%, respectively. Clone numbers by tissue are summarized in Table I.

**Table I.** Number of unique clones by tissue

	Peripheral Blood (P)	Normal Adjacent (N)	Tumor (T)
Dual TCRα	1,162	2,938	5,986
Dual TCRβ	348	1,206	2,301
Single TCR	8,437	25,145	45,010

Using the clone sizes calculated by the original authors using STARTRAC, we compared clonal expansion of dual TCR-expressing cells to single TCR-expressing cells. In peripheral blood, the mean clone sizes were nearly equivalent, regardless of TCR allele count. In contrast, the mean clone sizes of dual TCR-expressing cells were significantly larger (∼1.5- to 4-fold for dual *TRAV* and dual *TRBV*, respectively) than the mean clone sizes of single TCR-expressing cells in tissues (Figure 1C). Data were then split into CD4^+^ and CD8^+^ T cell subsets and compared by expressed TCR allele count in blood (Figure 1D) or combined tumor and normal tumor-adjacent tissues (Figure 1E). As expected, CD8^+^ T cells in all groups were more expanded than CD4^+^ T cells. In peripheral blood, the amount of expansion was similar for dual TCR-expressing cells and single TCR-expressing cells. In tissue, however, the mean clone size in dual *TRAV*- and dual *TRBV*-expressing CD8^+^ cells was significantly larger than single TCR-expressing CD8^+^ T cells. Thus, dual TCR-expressing clones are expanded to a greater extent, on average, in tissues, regardless of whether the tissue is tumor or normal adjacent tissue. This observation highlights the importance of collecting multiple samples per individual, because comparison of only tumor tissue with peripheral blood would have misled to the conclusion that dual TCR cells expand in a specific response to tumors. It would be interesting to compare normal tissue from healthy donors with tumor tissue to further resolve whether dual TCR cells preferentially accumulate in tissue or whether tissues must be inflamed for this to occur.

**FIGURE 2.**
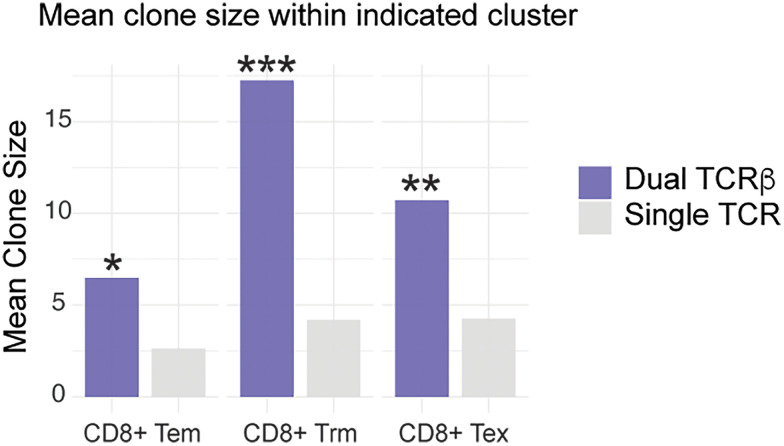
Clonal expansion by T cell phenotype. Initial analysis indicated that dual TCR CD8^+^ clones were more expanded (larger mean clone size) in tissue than single TCR clones. To determine whether certain CD8^+^ T cell clusters were differentially expanded, the mean clone size was calculated for dual and single TCR clones for each cluster. Clusters with significantly different mean clone size greater than twofold include early effector memory (Tem), tissue-resident memory (Trm), and terminally exhausted (Tex) phenotypes. Thus, CD8^+^ T cells with dual TCR expression may have greater ability to differentiate into tissue-resident cells, clonally expand within tissue, or migrate into tissues and be retained there. Significance was determined by Welch’s ANOVA with Games-Howell correction (*p < 0.05, **p < 0.01, ***p < 0.001).

Expression of dual TCRs may allow dual specificity, and it is interesting to note that the total number of TCRs on a single cell’s surface is constant between single and dual TCR-expressing cells (33). This could have an important bearing on TCR signal strength if only one of the two receptors is engaged, which in turn heavily influences postactivation phenotypes such as cytotoxicity, memory, or exhaustion (42). In viral models, dual expressors formed memory more efficiently (33). Zheng et al. used Seurat to cluster cells and assign cluster identities on the basis of gene expression (40), and we compared the level of clonal expansion of dual TCR-expressing cells between phenotypic subsets. Interestingly, dual *TRBV*-expressing CD8^+^ T cells with effector memory (GSMK^+^ early Tem), tissue-resident memory (ZNF683^+^CXCR6^+^ Trm), and terminally exhausted (terminal Tex) phenotypes were significantly more expanded than single TCR-expressing cells of the same phenotype (Figure 2). Overrepresentation of dual expressors in memory subsets is consistent with the viral infection literature; however, the observation that normal adjacent tissue and tumor tissue are highly similar might suggest that general inflammation or tissue homing rather than tumor Ag specificity drives the accumulation of expanded dual expressors.

A subset of T cells called mucosal-associated invariant T (MAIT) cells are often found in tissues and were recently observed to express dual *TRAV* at high frequencies (43). These dual-expressing MAIT cells exhibited preferential TCR gene use (e.g., high use of *TRAV1-2*). To determine whether TCR gene use is skewed in the cancer cohort, we plotted *TRAV* and *TRBV* gene use in unique clones expressing dual *TRAV*, dual *TRBV*, or single TCRs. *TRAV* exon use was nearly identical across allele groups. *TRBV* exon use was also nearly identical, with the notable exception that dual *TRBV*-expressing cells exhibit much higher use of *TRBV21-1*. *TRBV21-1* is a pseudogene (unlikely to produce a translated protein), and it is possible that *TRBV21-1* represents a first and unsuccessful *TRBV* rearrangement before moving on to rearrange the second allele. Nevertheless, it is interesting to note that the rearranged allele including the *TRBV21-1* gene continues to be transcribed. This could be consistent with mouse models where use of rare *Trbv* genes was skewed in cells with the capability of expressing two rearranged *Trbv* alleles compared with cells that expressed only a single *Trbv* (24). The minimal difference in gene use distribution between allele groups and a gene expression program inconsistent with MAIT cells leads us to conclude that dual expressors in human cancer tissues are not an invariant cell type.

## Open Questions for Single-Cell TCR Repertoire Profiling

Engineering a tumor-reactive TCR into cells retaining endogenous TCR expression for adoptive transfer therapy revealed an interesting phenomenon by which the α- and β-chains can mix and match, yielding up to four distinct dimers (37). Importantly, the total number of TCRs an individual T cell expresses on its surface appears constant, regardless of how many TCR αβ dimers are produced (33). Thus, dual TCR expression at the T cell surface would effectively reduce the absolute number of each individual TCR αβ dimer. This could profoundly impact Ag-specific T cell signaling.

It is well appreciated that TCR signal strength determines the activation phenotype of T cells (44). Signal strength is a function of the affinity and avidity of the T cell interaction with the APC (45, 46). “Affinity” is defined as the strength of the interaction between a single TCR molecule and a single peptide-bound MHC. “Avidity” is the cumulative effect of the total number of TCRs engaged plus the engagement of additional coreceptors. Whether a T cell becomes activated is an integration of affinity, avidity, and epitope density. If above the signal threshold, the magnitude of T cell activation is also directly impacted by these same properties. In the TME, high-affinity interactions contribute to T cell dysfunction, whereas low-affinity interactions lead to functional inertness, such that dialing in the optimum, intermediate signal strength is important to maximize antitumor activity (47).

The proportion of each TCR on the cell surface of a dual TCR-expressing cell also depends on post-translational allelic exclusion mechanisms by which dual surface expression is lower for one of the dimers (11). Two models have been posited to explain phenotypic allelic exclusion: competition and selective retention. The competition model describes a scenario in which cells with dual α-chains compete for pairing to a single β-chain and/or CD3 (48, 49). The α-chain with higher affinity for the β-chain and/or CD3 is preferentially incorporated into complete TCRs to be expressed at the cell surface, excluding the α-chain with lower affinity. Interestingly, competition may not be as strong in cells expressing one α and two β alleles, further distinguishing mechanisms of α and β exclusion (50). The selective retention model suggests that TCRs that recognize and appropriately bind self-MHC are retained on the cell surface because of signal propagation that prevents internalization (49, 51, 52). This model proposes that non-MHC restricted αβ pairs are endocytosed and degraded, excluding them from the cell surface. It is likely that dual TCR surface expression is regulated by several interacting processes. Nonetheless, the imperfect concordance between dual mRNA expression and protein expression of two functional TCRs on the cell surface complicates the study of an already small population of cells.

On the basis of the current understanding of mechanisms governing surface TCR expression, it is reasonable to hypothesize that the composition of the TCR pool on a cell expressing multiple alleles will be highly clone specific. Moreover, the relative proportions of TCR composition could exhibit plasticity based on factors such as temporal fluctuation in cognate Ag expression. Observing TCR pool composition over time will be impossible without single-cell surfaceomics. Nevertheless, it will be very important to understand these mechanisms to predict the functional consequences of dual TCR expression.

**FIGURE 3.**
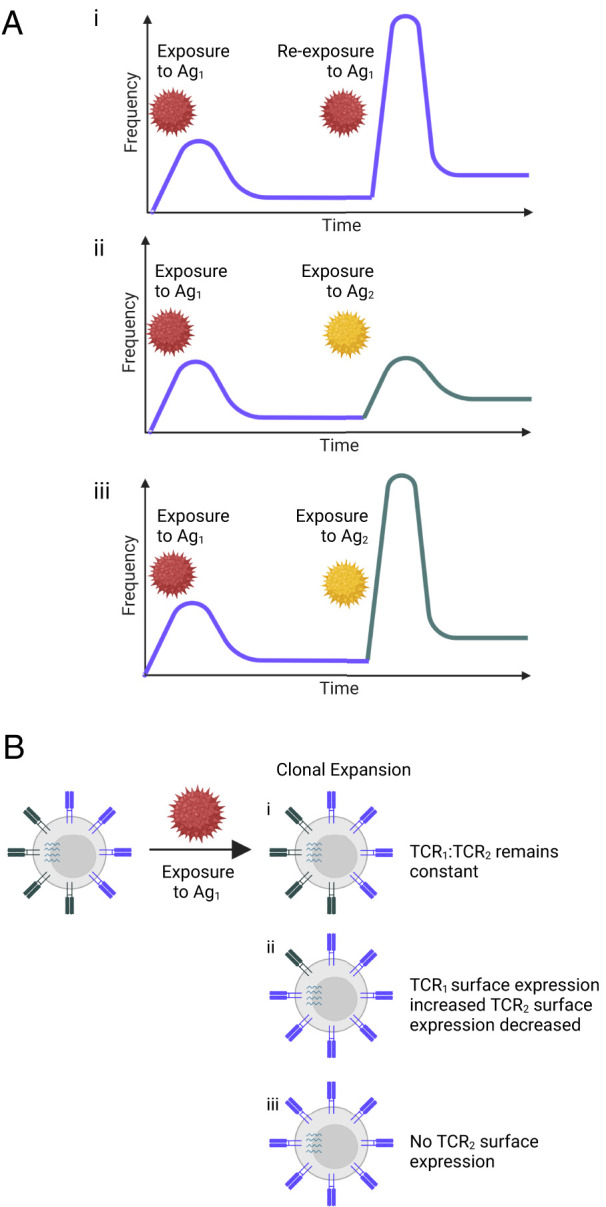
Open questions: dual TCR expressors over time. (**A**) Dual reactive T cells may respond to sequential Ag exposure in unique ways, and it is unclear whether primary exposure to the secondary Ag would induce a primary or memory response. (**i**) A single TCR T cell expands in response to Ag (Ag_1_, red) exposure, then contracts and forms memory; secondary exposure to the same Ag launches a stronger, more rapid response. Dual cells may differ in response to sequential Ag exposure. Two possibilities include (**ii**) Ag_1_ memory cells exposed to a second Ag (Ag_2_, yellow) exhibit a response similar in time and magnitude to the primary response, and (**iii**) Ag_1_ memory cells exposed to Ag_2_ have been preprimed and exhibit a memory-like response. Figure created with BioRender.com. (**B**) After a dual TCR-expressing cell is activated by exposure to Ag (Ag_1_), expanded clones may differ in the proportion of TCR surface expression by virtue of post-translational allelic exclusion. Some possibilities include (**i**) the ratio of TCR_1_ (purple) and TCR_2_ (teal) remains constant, (**ii**) surface expression skews and expression of the activated TCR_1_ increases and expression of TCR_2_ decreases, (**iii**) only the activated TCR_1_ is expressed at the cell surface. Furthermore, these could coexist within the clonal pool. Figure created with BioRender.com.

Longitudinal analysis of dual reactive T cells, including naturally occurring clones and TCR or CAR engineered cells, as they are exposed to sequential Ag will also be important in understanding memory formation, because it seems that dual expressing cells may develop memory phenotypes more readily than single expressors. A hallmark of memory is that second exposure to the same Ag results in a stronger, faster T cell response. If a T cell has dual reactivity and responds to one Ag, does subsequent exposure to its second cognate Ag induce a memory response, or does the response resemble a primary response (Figure 3A)? Does exposure to Ag change surface composition (Figure 3B)? Our understanding of dual TCR-expressing cells and their functions within the immune repertoire will require exploration of these important questions using single-cell technologies.

## FUTURE DIRECTIONS

Reanalysis of existing data with particular focus on dual TCR-expressing cells is a low-cost and fairly simple process that may yield substantial, additional understanding of this relatively small cell population. In this review, we performed a reanalysis of an exceptionally high-quality human cancer single-cell sequencing dataset where the original authors did not acknowledge dual TCR-expressing cells (40). Consistent with prior literature in other pathologies, dual TCR-expressing cells were present in peripheral blood and in cancer and normal adjacent tissues. Again, consistent with prior literature, dual TCR-expressing cells were frequently of memory phenotypes.

Surprisingly, however, clonal expansion of dual TCR-expressing cells was greater than clonal expansion of single TCR-expressing cells in tissues, regardless of whether the tissue was the tumor itself or normal adjacent tissue. This suggests that perhaps dual expressors have an advantage for homing to inflamed tissues or better clonal expansion capabilities in situ. This potentially important observation would have been missed if samples only included tumor and peripheral blood. It would be interesting to know the Ag specificity of these expanded cells, because they may be autoreactive and not necessarily tumor Ag specific. Antigenicity of dual TCR cells becomes even more important to understand in the context of immunotherapy response, because immunotherapy immune-related toxicities are often autoimmune in nature (31).

Despite greater magnitude of clonal expansion, endogenous dual TCR expressors still represent a minority of the overall number of T cells in tissues and the periphery. It remains unclear whether dual expressors would have measurable physiologic impact on antitumor immunity. This minority is further diminished when considering that not all cells expressing dual *TRAV* or dual *TRBV* at the mRNA level express two TCRs at the protein level on the cell surface.

Understanding the complexity of the TME at a high granularity is extremely important because of the heterogeneous and ever-evolving nature of cancer. In addition to single-cell gene expression approaches, single-cell proteomic approaches are being developed, which will allow us to understand which TCR αβ dimers are expressed on the cell surface and in what proportions (53). Coupling that information with spatial and temporal resolution will make it possible to answer basic biological questions about sequential Ag exposure of dual reactive cells that simply cannot be evaluated at the population level. Future workflows for single-cell analyses should include dual TCR-expressing cells as part of TCR repertoire analyses.

## Code Availability

All data in this review were generated and made publicly available by the original authors, Zheng et al. (40). Data were downloaded from https://zenodo.org/record/5461803#.Y9fe4-xMH6Y, and the script generated for this reanalysis is available at https://github.com/RogersLabGroup/Dual-TCR-in-cancer.
